# Expanding the MYCN Variant Spectrum in Feingold Syndrome Type 1: A Novel N-Terminal Missense Variant Segregating in an Affected Family

**DOI:** 10.3390/genes17050552

**Published:** 2026-05-05

**Authors:** Francisco Javier Mérida De la Torre, Javier Porta Pelayo, Inmaculada Ortiz-Martín

**Affiliations:** 1 Genetics Laboratory, Hospital Regional Universitario, 29011 Málaga, Spain; 2 Genologica by Health in Code, 29016 Málaga, Spain; javier.porta@genologica.com (J.P.P.); inmaculada.ortiz@genologica.com (I.O.-M.)

**Keywords:** Feingold Syndrome Type I, MYCN, developmental disorder, congenital abnormalities, case report

## Abstract

This study reports a previously unreported heterozygous MYCN missense variant, c.454G>A (p.Ala152Thr), identified in a child and two affected relatives, with clinical findings consistent with Feingold syndrome type 1, an autosomal dominant developmental disorder most commonly caused by loss-of-function variants in MYCN. The proband presented with a cleft palate, craniofacial dysmorphism, feeding difficulties, hypotonia, and characteristic digital anomalies. Similar features were observed in the father and sibling. Clinical exome sequencing revealed the novel MYCN variant, which was confirmed by Sanger sequencing and demonstrated co-segregation with the phenotype. Although most pathogenic MYCN variants leading to FS1 truncate the protein, this missense change lies within the N-terminal transactivation domain, a region involved in transcriptional regulation and protein stability. The physicochemical alteration introduced at residue Ala152 may plausibly affect MYCN function, consistent with haploinsufficiency as the established disease mechanism. According to the 2024 ACGS Best Practice Guidelines, the variant was classified as a variant of uncertain significance leaning toward pathogenicity. This report expands the mutational spectrum of MYCN, supports the potential clinical relevance of N-terminal missense variation in MYCN, and highlights intrafamilial phenotypic variability in FS1.

## 1. Introduction

Feingold Syndrome (FS) is a rare autosomal dominant developmental disorder first described in 1975, characterized by microcephaly, digital anomalies, craniofacial dysmorphism, gastrointestinal (GI) atresia, and mild-to-moderate intellectual disability. Core manifestations include brachymesophalangy of the second and fifth digits, fifth finger clinodactyly, toe syndactyly, and microcephaly. Historically, FS has also been referred to as ODED, MODED, and MMT syndromes (MIM#164280). Penetrance is generally complete with markedly variable expressivity, and approximately 60% of cases are inherited [1,2,3,4,5,6,7,8,9].

Two molecularly distinct forms are recognized (Table 1). FS type 1 (FS1, MIM#164280) is caused by loss-of-function (LoF) variants in MYCN (MIM*164840) at 2p24.3. MYCN encodes N-Myc, a transcription factor of the MYC family, essential for the embryonic development of the nervous system, limbs, and viscera [5,10,11]. The canonical protein, encoded by exons 2 and 3, contains C-terminal bHLH and leucine-zipper domains required for dimerization and DNA binding. FS1 results from MYCN haploinsufficiency, most commonly due to nonsense or frameshift variants that truncate the protein and disrupt these domains [5,6].

In contrast, gain-of-function (GoF) missense variants in MYCN cause Megalencephaly-Polydactyly Syndrome (MPS, MIM#620748), a phenotypic mirror of FS1 characterized by megalencephaly and postaxial polydactyly. These variants (e.g., T58M, P60Leu) impair Thr58 phosphorylation, increasing protein stability [12].

FS type 2 (FS2, MIM#614326) results from the heterozygous deletion of 13q31.3, including MIR17HG, leading to miR-17~92 haploinsufficiency [13,14]. Although FS1 and FS2 overlap clinically, they involve distinct molecular pathways [4,15].

In this work, we identify the novel heterozygous missense MYCN variant c.454G>A (p.Ala152Thr), classified as VUS-warm, segregating in a family with FS1 features, expanding the MYCN variant spectrum and contributing to genotype–phenotype refinement.

## 2. Materials and Methods

### 2.1. Clinical Data

The proband and first-degree relatives were evaluated at the Genetics Department of Málaga Regional University Hospital. Clinical assessment included physical examination and detailed personal and family history. Written informed consent for participation and publication was obtained. Ethical review and approval were waived for this study as per local legislation. Approval from the ethics committee is not required for standard clinical practice; however, the committee was informed. The patients and their legal representatives were asked to provide authorization for the publication of their medical data and relevant photographs through informed consent. All procedures adhered to ethical standards and the Declaration of Helsinki.

### 2.2. Genomic DNA (gDNA) Extraction

Genomic DNA was extracted from peripheral blood (Magna Pure 24, Roche Diagnostics. Basel, Switzerland). The DNA quantity and quality were assessed using Qubit 3.0 and NanoDrop ND-2000, and the DNA integrity and purity were evaluated based on 260/280 and 260/230 absorbance ratios.

### 2.3. Sanger Sequencing Analysis

Exon 2 of MYCN was amplified via PCR and sequenced using automated Sanger sequencing with M13-tagged primers. The sequences were aligned to NM_005378.6 with Mutation Surveyor software v.3.20. The primer sequences were MYCN_2F (Forward): 5′-GTAAAACGACGGCCAGTctgcatgtggagcggcttc-3′ and MYCN_2R (Reverse): 5′-AGCGGATAACAATTTCACACAGGgccaagacatacgagcactaacaaa-3′.

### 2.4. Clinical Exome Sequencing

Whole-exome sequencing was performed using a target enrichment method based on capture with specific probes (KAPA HyperExome V2; Roche Diagnostics), followed by paired-end massively parallel sequencing on a DNBseq-G400 platform (MGI Genomics).

### 2.5. Bioinformatic Analysis

Sequence data analysis was performed using the GenoSystem Variant Analysis software. This software, developed by Genologica S.L., incorporates an optimized algorithm that includes, among other steps: (a) initial quality control of the sequencing data; (b) sequence filtering to remove ambiguous bases, adapter sequences, and low-quality regions; (c) a second quality control step; (d) alignment to the hg19 reference genome; (e) variant and copy number variation (CNV) calling; (f) assessment of mapping coverage; and (g) variant annotation.

The software is specifically designed to identify variants located within exonic and splice-site regions (±10 bp) with an allelic frequency greater than 30% of sequencing reads. CNV analysis was performed using the ExomeDepth software.

Variant classification followed the ACMG/AMP guidelines with ACGS 2024 refinements [15,16] and was reviewed by a multidisciplinary team in collaboration with the referring clinician in order to verify fulfillment of the classification criteria.

## 3. Results

### 3.1. Clinical Evaluation

The proband was a three-year-old female referred for an incomplete cleft palate. She was born at 39 + 6 weeks (birth weight 2600 g; head circumference 35 cm, 35th percentile), after an uncomplicated pregnancy. She presented with feeding difficulties requiring nasogastric tube feeding. Newborn metabolic screening was normal.

**Figure 1:**
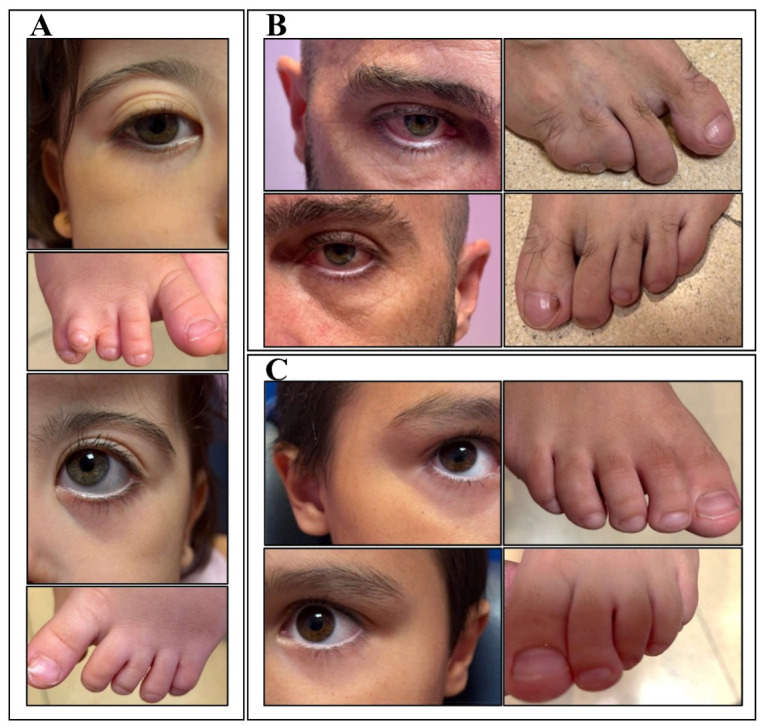
Overlapping physical features. (**A**) From top to bottom: right eye, right foot, left eye, and left foot of the proband. (**B**) and (**C**) show, from right to left, the right eye, right foot, left eye, and left foot of the father and sibling, respectively.

Examination revealed an incomplete cleft palate and craniofacial dysmorphism, including hypotelorism, low nasal bridge, epicanthal folds, bilateral orbital insufficiency with proptosis, anteverted nares, and retrognathia. Additional findings included widely spaced nipples and syndactyly of the third, fourth, and fifth toes of the right foot with hypoplasia of the fourth toe (Figure 1). The neurological examination revealed mild axial hypotonia and intermittent strabismus; deep tendon reflexes were normal. The transfontanellar ultrasound was normal.

The family history revealed that the father has a complete cleft palate, a short stature treated with growth hormones, and syndactyly. The six-year-old sibling presents with syndactyly and milder dysmorphic features than her sister (hypotelorism, epicanthal folds, and bilateral orbital insufficiency with proptosis) (Figure 1). The mother is asymptomatic. No additional congenital anomalies have been reported among relatives.

### 3.2. Genetic Analyses

**Figure 2:**
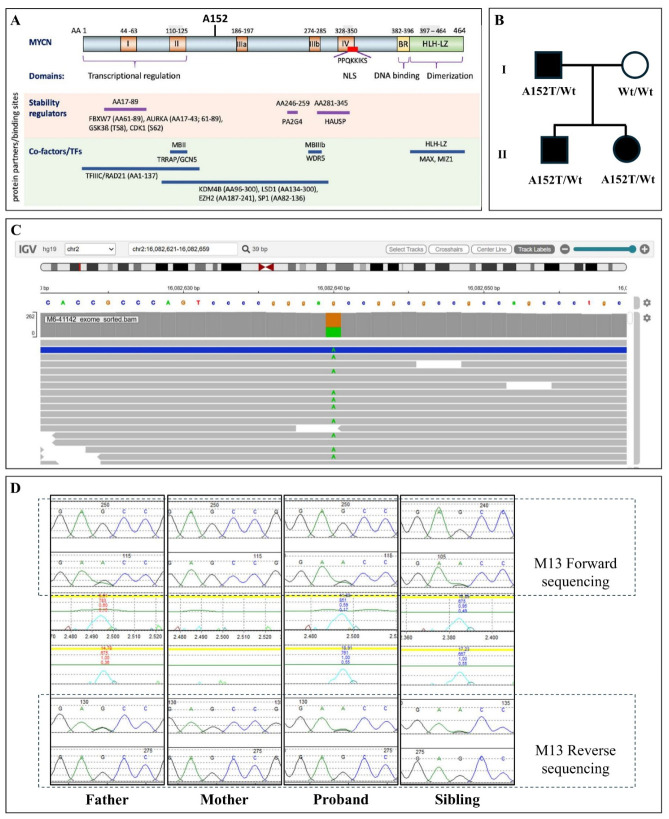
Results of genetic analyses. (**A**) The structure and functional domains of MYCN, showing the position of the A152T variant (adapted from Liu et al., 2021 [17]). (**B**) Pedigree of the family showing the affected proband (II-2). The father (I-1) and sibling (II-1) are also affected, while the mother (I-2) is unaffected with the wild-type alleles. (**C**) Integrative Genomics Viewer (IGV) image from whole-exome sequencing (WES) showing the heterozygous missense variant at the MYCN gene on chromosome 2 (Chr2:16082640, hg19). (**D**) The result of a family segregation study using Sanger sequencing confirms the presence of a heterozygous missense mutation (c.454G>A; p.Ala152Thr) in the proband, their father, and their sibling and its absence in their mother.

WES identified a previously unreported heterozygous MYCN variant, c.454G>A (p.Ala152Thr), in exon 2 (NM_005378.6) (Table 2, Figure 2C). Sanger sequencing confirmed the variant and demonstrated co-segregation in two informative meioses (PP1_supporting). The variant substitutes alanine with threonine at position 152, which is a low to weak-moderate conserved residue located in the N-terminal transactivation domain near Myc boxes (Figure 2A). The variant is reported in 1 of 1431958 alleles from gnomAD v.4.1.0 (PM2_supporting) and the phenotype is highly specific to MYCN-related Feingold syndrome (PP4_supporting), while in silico analyses support BP4 (supporting benign) (Table 3). Integration of these criteria within the ACGS Bayesian framework did not reach the posterior probability threshold for likely pathogenic classification. The variant was therefore classified as a Variant of Uncertain Significance (VUS); however, we use the term VUS-warm to denote increased evidence toward pathogenicity than toward benignity.

Additional complementary studies provided by the proband (karyotype and 180K array CGH) did not show structural or numerical abnormalities.

## 4. Discussion

The novel MYCN p.Ala152Thr variant lies within the N-terminal regulatory region, outside the canonical bHLH–LeuZ domains. Although most FS1-associated variants are truncating, missense variants affecting regulatory regions have also been reported, particularly when they affect regions essential for protein stability, transcriptional regulation, or post-translational modification [6,17,18,19,20].

The N-terminal region of MYCN contains regulatory elements that govern protein turnover, including phosphorylation sites and motifs that modulate proteasomal degradation. Although Ala152 is not part of a known phosphorylation consensus sequence, its location places it near regions critical for maintaining the structural configuration required for proper targeting to ubiquitin-mediated degradation pathways [17]. The substitution introduces a polar residue capable of hydrogen bonding, which could potentially influence local protein properties, such as conformation or interaction interfaces. This change might have implications for transcriptional activity or protein stability; however, functional studies would be required to determine its molecular impact.

The segregation of the variant with the phenotype in the proband, father, and sibling provides supportive evidence for its clinical relevance. All the affected individuals exhibit features consistent with FS1; these findings are compatible with reduced MYCN function during embryogenesis, consistent with the established haploinsufficiency mechanism. The absence of the variant from population databases supports its rarity, although this alone does not establish pathogenicity. Although in silico tools predict a tolerated effect, these algorithms may underestimate the pathogenicity in regions where structural changes have subtle but functionally relevant consequences. Indeed, several pathogenic MYCN missense variants reported in FS1 involve residues outside the C-terminal domains and exert their effect through impairment of transcriptional function rather than through gross structural disruption.

The phenotypic presentation in this family also highlights the variable expressivity that characterizes FS1. The proband presents more pronounced craniofacial features and feeding difficulties, while the father shows a milder but clearly overlapping spectrum. Such intra-familial variability is well documented in FS1 and likely reflects modifier genes or environmental influences that modulate the impact of MYCN haploinsufficiency.

The variant described in this work could differ mechanistically from the gain-of-function (GoF) missense variants associated with MPS, which cluster around residues critical for MYCN phosphorylation and stabilization (e.g., Thr58, Pro60). These GoF variants enhance protein stability and activity, leading to clinical manifestations that are inverse to those observed in FS1. In contrast, the p.Ala152Thr variant is located outside this well-characterized hotspot and does not overlap residues known to mediate MYCN stabilization, suggesting a molecular mechanism distinct from the established GoF variants. However, the functional consequences of this substitution cannot be inferred solely from its position and would require experimental validation.

A major strength of this study is the comprehensive clinical, familial, and molecular characterisation of the proband and affected relatives, which allows a genotype–phenotype correlation and a careful evaluation of the potential contribution of the identified variant to the observed clinical features. The detailed phenotypic description and segregation analysis provide valuable data that may facilitate future comparisons and contribute to the collective interpretation of rare variants. The main limitation of this work is its focus on a single family, which restricts the ability to draw definitive conclusions regarding causality. In addition, the reported variant is currently classified as a variant of uncertain significance (VUS), reflecting the limited available evidence at present. Nevertheless, the publication of well-documented cases such as this one is essential to support data sharing, enable future reclassification as new evidence emerges, and advance knowledge in rare genetic disorders.

## Figures and Tables

**Table 1:** Clinical and Genetic Features of Feingold Syndrome Type 1 (FS1) vs. Type 2 (FS2).

Feature	FS1	FS2
**Gene involved**	MYCN (loss-of-function: missense, nonsense, frameshift mutations, deletions)	MIR17HG (deletions or variants affecting the miRNA cluster)
**Digital anomalies**	Brachymesophalangy: **94–100%**; Toe syndactyly: **43–97%**; Thumb hypoplasia: **14–17%** (PMID: 18470948, 30088856, 10905665)	Brachymesophalangy: **100%** (PMID: 26360630, 30672094)
**Microcephaly**	**70–89%** (PMID: 18470948, 26360630, 30088856)	**88–89%** (PMID: 26360630, 30672094)
**Gastrointestinal atresia (GA)**	**24–55%**; EA/TEF up to **35%**; DA up to **27%** (PMID: 18470948, 10905665, 26360630)	**Absent** (PMID: 26360630, 30672094)
**Neurocognitive**	Developmental delay/Intellectual disability: **45–71%**; severe ID possible; white matter T2 hyperintensities reported (PMID: 18470948, 30088856, 33442900)	ID/Learning disability: **100%** (PMID: 30672094)
**Short stature**	**56–60%** (PMID: 18470948, 30088856)	**86%** (PMID: 30672094)
**Cardiac anomalies**	**15–30%** (PMID: 18470948, 30088856)	**40%** (PMID: 26360630, 30672094)
**Renal anomalies**	**18–29%** (PMID: 18470948, 30088856)	Not commonly reported
**Hearing loss**	**7–43%** (PMID: 18470948, 30088856, 10664710)	**66%** (PMID: 30672094)
**Other features**	Vertebral anomalies, laryngeal cleft, congenital absence of flexor pollicis longus tendon (PMID: 32925198)	Keratoconus, cognitive issues (memory impairment, insomnia) (PMID: 28159702, 26026879)

**Table 2:** Reported MYCN Pathogenic Variants and Associated Phenotypes.

Feingold Syndrome Type 1				
**ToV**	Localization	Mechanism	Reported variants	References (PMID)
Deletion	2p24.1- 2p24.3	HI	del2p24.2-p25.1 microdeletion in 2p23-p24 1.2Mb microdeletion DelFAM84A_MYCN; DelMYCNOS_MYCN exon2; DelFAM84A_MYCN; DelMYCNOS_MYCN 425 Kb del 2p24.3 2p 3.4-16.8 deletions 4.4 Mb microdeletion in 2p24.3-p24.2 del2p24.3; del2p24.3-p24.2	[8989454] [10677303] [15821734] [18470948] [21224895] [22842076] [30088856] [33442900]
onsense/Frameshift	Exons 2/3	LoF	c.626dupC (p.A319Gfs)	[18671284]
			c.134dupC (p.E47fs); c.217G>T (p.E73 *); c.231G>A (p.W77 *); c.302delG (p.G101fs); c.451G>T (p.G151 *); c.662C>A (p.S221X); c.683delC (p.P228fs); c.836_837dup (p.V280fs); c.881_882dup (p.T295fs); c.915_916insT (p.A306fs); c.964C>T (p.R322 *); c.1005delC (p.S336fs); c.1097dupA (p.A367fs); c.1105_1106dup (p.S369fs); c.1117C>T (p.R373 *); c.1207delA (p.T403fs); c.1274dupA (p.A426fs); c.1293delC (p.S432fs); c.1338delA (p.K446fs)	[18470948]
			c.1110insG (p.S371Efs); c.928-930insGT (p.P310Rfs); c.474-514del (p.G161Pfs)	[21224895]
			c.503_543del (p.A171Rfs); c.1117C>T (p.Arg373 *); c.1168 G>T (p.E390 *)	[33442900]
			c.266dupG (p.S90Qfs)	[34926353]
Missense	Exon 3	LoF	c.1145G>A (p.R382H) c.1177C>A (p.R393S); c.1178G>A (p.R393H); c.1181G>A (p.R394H); c.1226C>T (p.P409L) c.1177C>T (p.R393C) c.1171C>T (p.R391C)	[18671284] [18470948] [21224895] [32925198]
**Megalencephaly-Polydactyly Syndrome**				
**ToV**	**Localization**	**Mechanism**	**Reported variants**	**References**
Missense	Exon 2	GoF	c.173C>T (p.T58M) c.179C>T (p.P60L)	[30573562] [37710961]

**Table 3:** Evaluation of ACMG/AMP criteria supporting the classification of the MYCN variant.

Evidence	Justification	ACMG 2015 ClinGen Considerations	Scores
Very low frequency in gnomAD v4.1.0	Extremely rare variant in population databases and compatible with a rare autosomal dominant condition	PM2_supporting	1/1,431,958 alleles No homozygotes
Cosegregation with disease	Limited but consistent within the family	PP1_supporting	2 informative meioses
Phenotype consistent with FS1	The patient phenotype is highly specific for FS1, MYCN haploinsufficiency (HI) is a known disease mechanism	PP4_supporting	HI score = 3 (Moderate)
In silico tools predict a benign effect	Concordant computational evidence supports a benign effect on protein function	BP4_supporting	REVEL: 0.018 AlphaMissense: 0.074 PolyPhen-2: 0.02
Low to weak-moderate evolutionary conservation	Insufficient to support PP3	Not applied	PhyloP100way: 1.235 GERP++: 0.118

## Data Availability

Data sharing is not applicable to this article, as no publicly available datasets were generated or analyzed in this study.

## References

[1] Marcelis C.L.M., de Brouwer A.P.M., Adam M.P., Bick S., Mirzaa G.M., Pagon R.A., Wallace S.E., Amemiya A. (2009). Feingold Syndrome 1. GeneReviews^®^.

[2] Brunner H.G., Winter R.M. (1991). Autosomal dominant inheritance of abnormalities of the hands and feet with short palpebral fissures, variable microcephaly with learning disability, and oesophageal/duodenal atresia. J. Med. Genet..

[3] Feingold M., Hall B.D., Lacassie Y., Martínez-Frías M.L. (1997). Syndrome of microcephaly, facial and hand abnormalities, tracheoesophageal fistula, duodenal atresia, and developmental delay. Am. J. Med. Genet..

[4] Celli J., van Beusekom E., Hennekam R.C.M., Gallardo M.E., Smeets D.F.C.M., de Córdoba S.R., Innis J.W., Frydman M., König R., Kingston H. (2000). Familial syndromic esophageal atresia maps to 2p23-p24. Am. J. Hum. Genet..

[5] van Bokhoven H., Celli J., van Reeuwijk J., Rinne T., Glaudemans B., van Beusekom E., Rieu P., Newbury-Ecob R.A., Chiang C., Brunner H.G. (2005). MYCN haploinsufficiency is associated with reduced brain size and intestinal atresias in Feingold syndrome. Nat. Genet..

[6] Marcelis C.L.M., Hol F.A., Graham G.E., Rieu P.N.M.A., Kellermayer R., Meijer R.P.P., Lugtenberg D., Scheffer H., van Bokhoven H., Brunner H.G. (2008). Genotype-phenotype correlations in MYCN-related Feingold syndrome. Hum. Mutat..

[7] Burnside R.D., Molinari S., Botti C., Brooks S.S., Chung W.K., Mehta L., Schwartz S., Papenhausen P. (2018). Features of Feingold syndrome 1 dominate in subjects with 2p deletions including MYCN. Am. J. Med. Genet. Part A.

[8] Muriello M., Kim A.Y., Sondergaard Schatz K., Beck N., Gunay-Aygun M., Hoover-Fong J.E. (2019). Growth hormone deficiency, aortic dilation, and neurocognitive issues in Feingold syndrome 2. Am. J. Med. Genet. Part A.

[9] Zeka N., Bejiqi R., Gerguri A., Zogaj L., Jashari H. (2022). A new variant of MYCN gene as a cause of Feingold syndrome. Clin. Case Rep..

[10] Ruiz-Pérez M.V., Henley A.B., Arsenian-Henriksson M. (2017). The MYCN Protein in Health and Disease. Genes.

[11] Ota S., Zhou Z.Q., Keene D.R., Knoepfler P., Hurlin P.J. (2007). Activities of N-Myc in the developing limb link control of skeletal size with digit separation. Development.

[12] Nishio Y., Kato K., Tran Mau-Them F., Futagawa H., Quélin C., Masuda S., Vitobello A., Otsuji S., Shawki H.H., Oishi H. (2023). Gain-of-function MYCN causes a megalencephaly-polydactyly syndrome manifesting mirror phenotypes of Feingold syndrome. HGG Adv..

[13] de Pontual L., Yao E., Callier P., Faivre L., Drouin V., Cariou S., Van Haeringen A., Geneviève D., Goldenberg A., Oufadem M. (2011). Germline deletion of the miR-17~92 cluster causes skeletal and growth defects in humans. Nat. Genet..

[14] Tassano E., Di Rocco M., Signa S., Gimelli G. (2013). De novo 13q31.1–q32.1 interstitial deletion encompassing the miR-17-92 cluster in a patient with Feingold syndrome-2. Am. J. Med. Genet. Part A.

[15] Richards S., Aziz N., Bale S., Bick D., Das S., Gastier-Foster J., Grody W.W., Hegde M., Lyon E., Spector E. (2015). Standards and guidelines for the interpretation of sequence variants: A joint consensus recommendation of the American College of Medical Genetics and Genomics and the Association for Molecular Pathology. Genet. Med..

[16] Durkie M., Cassidy E.J., Berry I., Owens M., Turnbull C., Scott R.H., Taylor R.W., Deans Z.C., Ellard S., Baple E.L. (2024). ACGS Best Practice Guidelines for Variant Classification in Rare Disease 2024. Assoc. Clin. Genom. Sci..

[17] Liu Z., Chen S.S., Clarke S., Veschi V., Thiele C.J. (2021). Targeting *MYCN* in Pediatric and Adult Cancers. Front. Oncol..

[18] Blaumeiser B., Oehl-Jaschkowitz B., Borozdin W., Kohlhase J. (2008). Feingold syndrome associated with two novel MYCN mutations in sporadic and familial cases including monozygotic twins. Am. J. Med. Genet. Part A.

[19] Cognet M., Nougayrede A., Malan V., Callier P., Cretolle C., Faivre L., Genevieve D., Goldenberg A., Heron D., Mercier S. (2011). Dissection of the MYCN locus in Feingold syndrome and isolated oesophageal atresia. Eur. J. Hum. Genet..

[20] Peleg A., Kurolap A., Sagi-Dain L., Larom-Khan G., Adir V., Mory A., Paperna T., Shuldiner A.R., Gonzaga-Jauregui C., Adir N. (2021). A novel mutation in MYCN gene causing congenital absence of the flexor pollicis longus tendon as an unusual presentation of Feingold syndrome 1. Clin. Dysmorphol..

